# A memristor fingerprinting and characterisation methodology for hardware security

**DOI:** 10.1038/s41598-023-33051-z

**Published:** 2023-06-09

**Authors:** Callum Aitchison, Basel Halak, Alex Serb, Themis Prodromakis

**Affiliations:** 1 Electronics and Computer Science, University of Southampton, University Road, Southampton, SO17 1BJ UK; 2 Centre for Electronics Frontiers, Institute for Integrated Micro and Nano Systems, School of Engineering, The University of Edinburgh, Edinburgh, UK

**Keywords:** Electrical and electronic engineering, Electronic devices

## Abstract

The modern IC supply chain encompasses a large number of steps and manufacturers. In many applications it is critically important that chips are of the right quality and are assured to have been obtained from the legitimate supply chain. To this end, it is necessary to be able to uniquely identify systems to aid in supply chain tracking and quality assurance. Many identifiers, however, can be cloned onto counterfeit devices and are therefore untrustworthy. This paper proposes a methodology for using post-CMOS memristor devices as a fingerprint to uniquely identify ICs. To achieve this, memristors’ unique and variable I–V characteristics are exploited to produce a fingerprint that can be generally applicable to a wide variety of different memristor technologies and identifiable over time, even where cell retention is non-ideal. In doing so it aims to minimise the hardware required on-chip both to minimise cost and maximise the auditability of the system. The methodology is applied to a $\text {TiO}_x$ memristor technology, and shown to be able to identify cells in a set.

## Introduction

In security or safety-critical applications it is imperative that systems can be trusted. Allowing a counterfeit, tampered, or otherwise illegitimate chip into devices in some applications could have disastrous consequences. The emergence of counterfeit ICs is a real and present threat in the electronics industry^1^. A counterfeit chip may not perform to standard, resulting in subpar performance which could cause safety failures. Similarly, a tampered chip could be intentionally altered by a bad actor to perform incorrectly, or introduce security vulnerabilities. For a secure deployed system, devices in the field must also be securely and individually identifiable, as well as able to protect data with their own unique keys.

With internet of things (IoT) and other autonomous systems expected to become increasingly pervasive in high-security and safety-critical situations, it is of paramount importance that the hardware on which they rely can be trusted. All types of applications could be affected by tampering. Examples of applications range from critical infrastructure, where reliability must not be compromised by counterfeit chips, to defence, where an adversary could intentionally manipulate or compromise hardware. The applications for a securely identifiable fingerprint extends across the supply chain, for tracking authenticity after manufacturing, and for systems already deployed in the field.

Devices could be tampered in the supply chain, or after deployment. Ensuring that the identity of these devices is as they claim is an important step in maintaining the security of these systems. Many options for identifying chips currently exist, for example serial numbers and watermarking^2,3^. Serial numbers offer only a basic level of security and can be easily cloned by an attacker with minimal effort to claim the identity of a legitimate chip. A further anti-counterfeit method of watermarking may also be employed to identify functional clones that have not been legitimately produced^2^. To do so, an intentional adjustment may be inserted into a design as a watermark, and its presence can be checked later. This however has the downfall of requiring sophisticated analysis of the chip and not protecting against overproduction. One method of overcoming these issues is the use of a Physical Unclonable Function (PUF)^4,5^, an unclonable hardware function that gives a unique response due to intrinsic and uncontrollable variation. A PUF differs from a random number generator in that the random response it produces should remain the same as long as is required for identification, without explicitly storing the key. The most common, and commercially-available, version of this is an SRAM PUF^6–8^.

As new post-CMOS devices are introduced there is the opportunity to exploit them for identifying chips. Resistive RAM (RRAM) is one such technology, based on arrays of memristors, a memory device based on programming analogue resistances. These devices are CMOS-compatible and can be integrated on a die with other standard CMOS circuits^9–11^. Because memristor-based memories are low power and more compact than the equivalent SRAM^12^, these memories are ripe for exploitation in future applications^10,11^. As memristors are analogue devices, as opposed to digital SRAM devices, these offer new avenues for fingerprinting CMOS chips.

Memristors may be used in further security applications in the future, including in random number generators^13–15^ and cryptographic accelerators^16^. These have shown power efficiency improvements over standard CMOS approaches, offering particular advantages in embedded applications. By using memristors in these applications it may also be possible to reduce the number of side channels available for attack. For example, improvements in hardness against power analysis attacks has been demonstrated by combining memristors and CMOS logic using hybrid CMOS/memristor gates^17^. These developments may allow a complete secure computing system to be built using a hybrid CMOS-RRAM design, enabling RRAM to play a key role in future secure hardware.

Fingerprinting is an essential primitive for securely identifying chips. With memristive devices becoming available, new avenues exist for identification^18–22^. Previous work has been based on specific RRAM technologies, relying on crossbar memories, or reading memristors’ values at only a single voltage level when set to a specific state^18,19,22^. This work, however, is based on differential comparison across a range of read voltages using standalone memristors. Such an approach provides better noise immunity and improved ageing resilience^23^. By comparing memristors’ responses across a range of voltages, variation in the I–V response is exploited. This work aims to demonstrate a general characterisation and identification methodology for memristive devices, exploiting the variation in I–V response in a way which requires only a set of individual cells. By relying primarily on the shape of I–V curve variation, rather than the absolute resistance or switching, this methodology differentiates itself from existing memristor-based identification schemes. It also remains generally applicable to a range of memristor technologies with non-linear I–V curves. Only a small amount of on-chip area needs to be dedicated to the identifier, as only a small array of two-terminal memristors needs to be individually readable.

Individual contributions of this work are: (1) characterisation methodology, (2) a system architecture for fingerprinting memristor cells, (3) a comparative analysis with existing methods.

The paper is structured as follows: Firstly, the methodology is introduced starting with the general approach to identifying variability and the rationale, followed by the detailed process to extract this data. Secondly, a methodology for exploiting the variation data to identify a device is explored. Third, results for one memristor technology are tested and evaluated in detail. Finally, the implications of these results are discussed, including comparisons with existing methods.

## Fingerprinting

### Approach

When a memristor is formed and programmed the cell exhibits a varying I–V characteristic^24^. Similarly, variability can be observed cycle-to-cycle as the memristor is set and reset^14,25^. The disparity in the cells’ characteristics gives each cell a different behaviour, which can make it differentiable from other cells.

Depending on the memristor technology used, these characteristics may be different or varying in reliability over time and environmental conditions. Because of this, a methodology was developed to identify and visualise variation in I–V behaviour between different cells, with the aim to uniquely identify memories based on their unique combination of characteristics. Such a methodology will be generally applicable to any resistive memory with a variable, non-linear I–V behaviour and offers an additional source of entropy as compared to simpler methodologies involving the absolute resistances of the high resistance or low resistance states. Applications could also extend to device development, where a baseline model could be used to identify good or poor memristor behaviours with further applications in production testing.

It is worth noting that the presented methodology is appropriate for use both with standalone devices/arrays that can be evaluated with commercially available instrumentation tools^26,27^, as demonstrated in this paper, as well as with memristor/ReRAM technologies monolithically integrated onto CMOS. The latter would require both the physical integration of technologies as well as the integration of design kits and models^28,29^ and is a potential avenue for future work. In such an integrated design, trade-offs must be made between convenience and the potential for an attacker to include additional hidden logic with reduced audibility.

### Rationale

To identify only intrinsic device variation, any external differences introduced to the memrisor should be minimised. To achieve this, the same forming and programming methodology must be applied to all cells in an array equally. Although not directly tested, it may be possible to reset any introduced biases by cycling the memristor between its high resistance and low resistance states. Once the memristors have all been programmed to the same state, their sub-threshold I–Vs may be collected without affecting their state. Some previous work, partly dependent on the voltage-dependent resistance behaviour, has been proposed as a PUF^20^. This work depends indirectly on the non-linearity of cell conductances in a 3-dimensional crossbar array and requires a large, highly reliable, array with a specific on-chip hardware for comparing the responses rows of the array to a specific stimulus. In comparison, the proposed method is generally applicable to identify standalone memristors in smaller arrays. The advantage of the proposed approach is a high degree of transparency as the majority of the processing can be performed in a trusted off-chip environment, away from any supply chain risks. Additionally, since a simple design requires only individual memristors to be on-chip with characterisation performed off-chip, the power characteristic is highly deterministic (limited to the $I^2 R$ required to read the resistance) which can enable a high degree of trust against hidden on-chip hardware. To this end, a methodology was developed using the ArC One memristor characterisation platform to individually address memristor cells, form them and characterise their I–V responses and variation.

### Characterisation

#### Data collection

**Figure 1:**
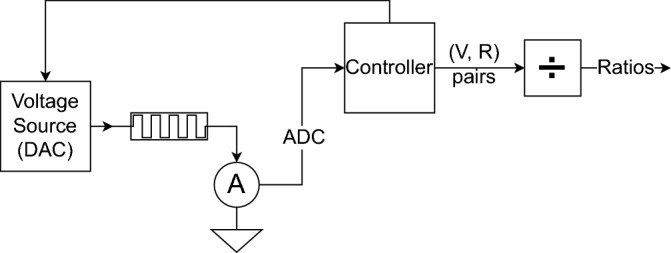
Proposed system architecture. A memristor is swept across voltages by the controller, and (V, R) pairs obtained for generation of a ratio matrix.

The data collection methodology is designed to approximate a hardware design, similar to that shown in Fig. 1. In this design a controller is capable of testing the memristor at arbitrary voltages via a DAC. The resulting current is then read back using an ADC. The amount of this design that may be incorporated on-chip can be varied to design requirements. In the extreme, the whole system could be implemented on-chip. This, however, negates many of the advantages in trust and auditability offered by completing identification using trusted known hardware. In addition, the on-chip area requirements are greatly increased. At the other extreme, only the memristors need to be be implemented on-chip. For such a design a large number of terminals will be required to individually access each memristor on the chip. By doing so, the maximum level of auditability is offered and minimal area is required. A realistic design may strike a compromise where only memristors and multiplexing logic are implemented on-chip, and a trusted off-chip device completes the data collection. Such a design would require minimal trust of the chip, whilst needing only a small amount of on-chip area by reducing the number of pads necessary for interfacing.

**Figure 2:**
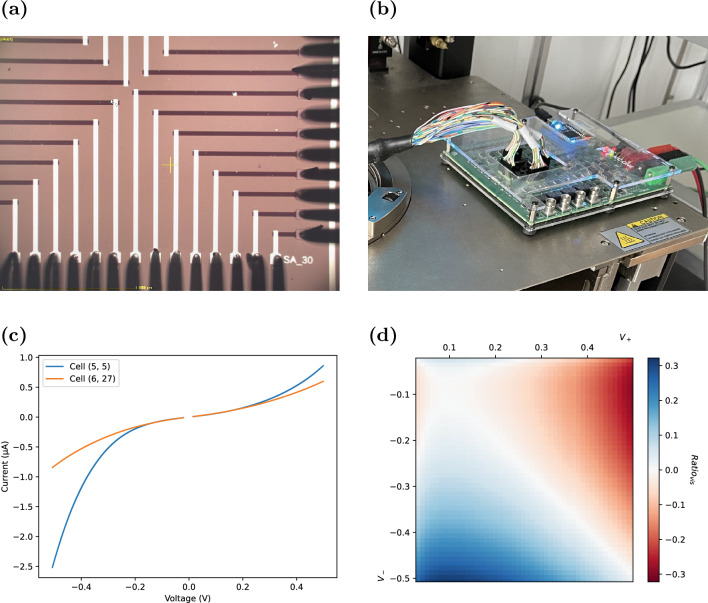
Characterisation experimental setup. (**a**) An array of standalone memristors (formed at the cross sections of the connecting wires) is probed with a probe card and individually addressed for characterisation by the (**b**) ArC One memristor characterisation platform. This data is then extracted as (**c**) I–V curves and then analysed in software to produce (**d**) a 2D matrix which can be plotted to visualise the magnitude of the ratio of resistances to positive compared to negative voltages.

The methodology was employed on 32-cell standalone memristor arrays. All cells in the array are treated equally from pristine (never previously used) to avoid incorporating any bias in the results. The cells were initially formed using the ArC One “FormFinder” tool, using a minimum voltage of 4*V* and a maximum of 9*V* with a 0.1*V* step, minimum pulse width of $100 \, \upmu s$ and maximum $1000 \, \upmu s$ with a $100 \%$ step, and $1 \, M \Omega$ resistance threshold. This gradually increases the voltage until a sub-$1 \, M \Omega$ resistance is achieved on read, indicating the device has successfully formed a conductive filament in the memristive active layer. After this, cells are repeatedly read with sub-threshold voltage ramps over $\pm 0.5 \, V$ with a $0.01 \, V$ step and 8*ms* pulse width. Each read cycle over the employed 32 cells takes approximately a minute, enabling a periodic measurement once per minute over a longer time period for averaging. The experimental setup for this process is shown in the top row of Fig. 2.

Data are gathered for an array over longer runs with an I–V characteristic measured for each cell over a period for short-term analysis initially (for example, 18 h) and at later intervals (days) for long-term analysis. The initial long run enables short-term changes after forming to be observed, whilst the latter runs allow the cells’ states to converge to help test the long-term reliability of the response.

#### Data analysis

Analysis of the data was based on comparing the ratio of the cells’ resistance to positive polaity voltages to the resistance to negative voltages. These ratios can be visualised as a 2D heat map after data collection, as shown in the bottom row of Fig. 2. For the purposes of producing a visualisation, the ratios were corrected according to the following formula:

1 $$\begin{aligned} Ratio_{vis} = {\left\{ \begin{array}{ll} + \left( \frac{R_+}{R_-} - 1\right) &{} R_+> R_- \\ - \left( \frac{R_-}{R_+} - 1\right) &{} R_- > R_+ \\ 0 &{} R_+ = R_- \end{array}\right. } \end{aligned}$$

where $R_-$ is the resistance to the negative voltage, and $R_+$ is the resistance to the positive voltage. Correcting the ratio in this way enables a linearly scaled heat map to clearly demonstrate the polarity and magnitude of the positive voltage resistance as compared to the negative voltage resistance.

By plotting these 2D heat maps for the cells some observations about variation and the quality of the response may be made visually. For example, for this technology a highly linear response may be indicative of a non-functional memristor. This will be visible in the visualisation as a blank or noisy plot of near-zero $Ratio_{vis}$ values. Whilst this is not a strict pass-fail test, such results may be quickly discounted as failed memristors, and are likely not to be useful as a fingerprint. Besides identifying the cells as non-functional, such cells offer little entropy for fingerprinting. Example plots demonstrating this situation have been included in the Supplementary Figs. 1–3.

In experimental data, even cells with similar resistances (as measured at $+0.2 \, V$) exhibit clearly different I–V responses, resulting in dissimilar resistance ratio plots.

These resistance ratio responses also appear stable over longer time periods. Whilst there is some shift in the absolute resistance at a given test voltage, the overall visual similarity between ratio plots remains strong. This is also fundamental to fingerprinting, as the signature must remain uniquely identifiable to generate a reproducible response. Although the plots still look similar, there is a reduction in the extent of the maximum ratios. This shows a tendency for the cells to become a little more linear over time, without substantially changing the pattern of the generated response. In most cases, with the tested memristor technology, this shift is not dramatic. Small shifts may be corrected by normalising the response.

By taking the ratio matrices used to produce the 2D plots, the Euclidean distance can be found between two different responses. For this to be used as a measure of uniqueness, ie whether a cell is unique from the others, the minimum Euclidean distance, Eq. (2), for each cell compared to every other cell (inter-ED) was compared to the value from the same cell (intra-ED) over an ageing period. If, after settling, a cell can be uniquely identified within the set it may be considered reliable. After ageing, a reliable cell will achieve a lower Euclidean distance to its original response than to any other response in the set. In cases where this does not produce acceptable results a “fuzzy” matching algorithm may be employed. By considering all cells with a Euclidean distance from the test cell within 25% of the minimum as candidate cells, the candidate cell with the closest resistance at 0.2*V* to the reference can be selected as the best match.

2 $$\begin{aligned} ED = \sqrt{\sum _{i=1}^{m} \sum _{j=1}^{n} |a_{ij} - R_{ij} |^2} \end{aligned}$$

where *a* is the test response matrix of $Ratio_{vis}$ values, and *R* is the reference cell response for comparison. The matrix dimensions in rows and columns (the number of $+\, V$ and $- \, V$ samples) are *m* and *n*, respectively.

### Results and evaluation

Devices were fabricated with e-beam evaporation for the metals and reactive magnetron sputtering for the active layer. Negative tone lithography and lift-off have been used throughout to define all the layers. First, the 12 nm thick Pt layer (bottom electrode) is deposited with e-beam evaporation using 5 nm of Ti as adhesion layer. Afterwards, a second lithography $\text {TiO}_x$ is deposited using reactive magnetron sputtering (Leybold Vacuum HELIOS) from a metallic Ti source in an oxygen/argon plasma (8 sccm $\text {O}_2$, 35 sccm Ar). The thickness of the active layer is 25 nm. The device is finalised with a further 15 nm Pt deposition to define the top electrode. The overlapping area defines the active device. Available device active areas range from 2 to $60 \, \upmu m^2$, and in this work the $5 \, \upmu m^2$ devices were used. Devices produced using this process are typically in the $G \Omega$ range and need to undergo an electroforming step prior to use.

**Figure 3:**
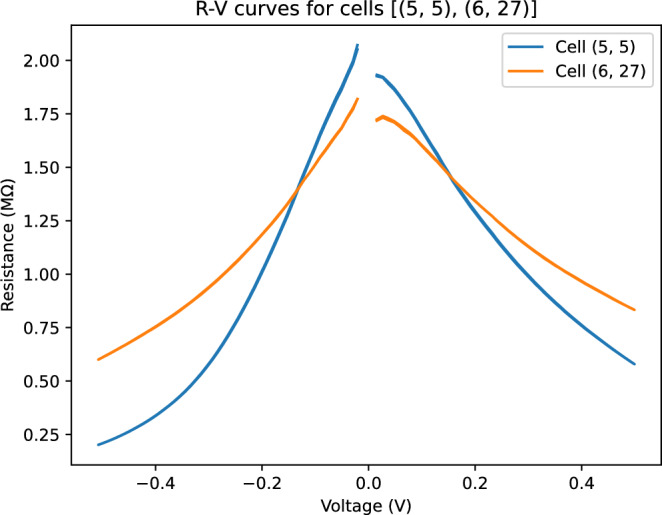
Raw V–R curves for two similar cells at coordinates 5, 5 (blue) and 6, 27 (orange).

The raw results for two similar cells is shown in Fig. 3. This shows the raw data retrieved from the cells and demonstrates the clear variation in resistance linarity for the memristor cells.

**Figure 4:**
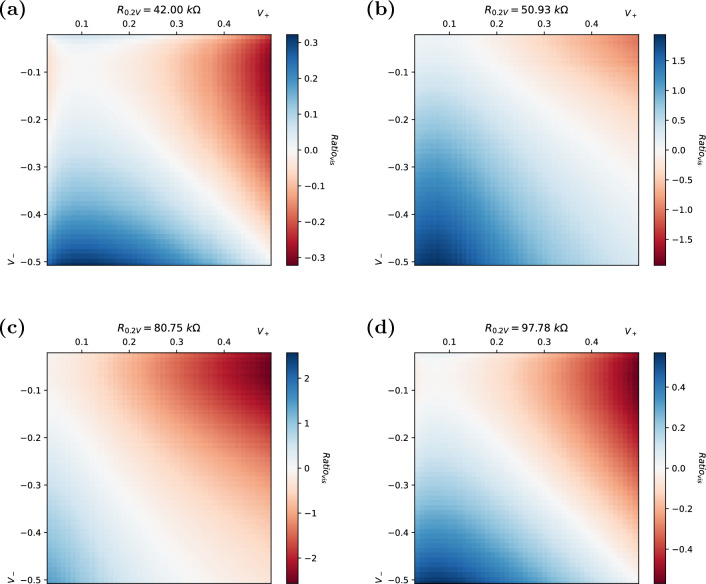
2D heat map of $Ratio_{vis}$ [Eq. (1)] for selected cells with similar resistances: (**a**) 22, 22 ($42\ k\Omega$), (**b**) 20, 20 ($51\ k\Omega$), (**c**) 17, 16 ($81\ k\Omega$), (**d**) 11, 11 ($98\ k\Omega$), measured at $+0.2 \, V$. Note individual scales.

As seen in the sample of plots shown in Fig. 4, even cells with similar resistances (as measured at $+0.2 \, V$, shown above plots) also exhibit clearly different 2D ratio responses. The figure shows the responses of four individual memristor cells from a 32-cell array when the $Ratio_{vis}$ is plotted as a heat map. The top two cells (22, 22 and 20, 20) and bottom two cells (17, 16 and 11, 11) have a particularly similar resistance but evidently different ratio plots. This demonstrates quite clearly that the cells have individual I–V signatures which can be targeted for fingerprinting.

**Figure 5:**
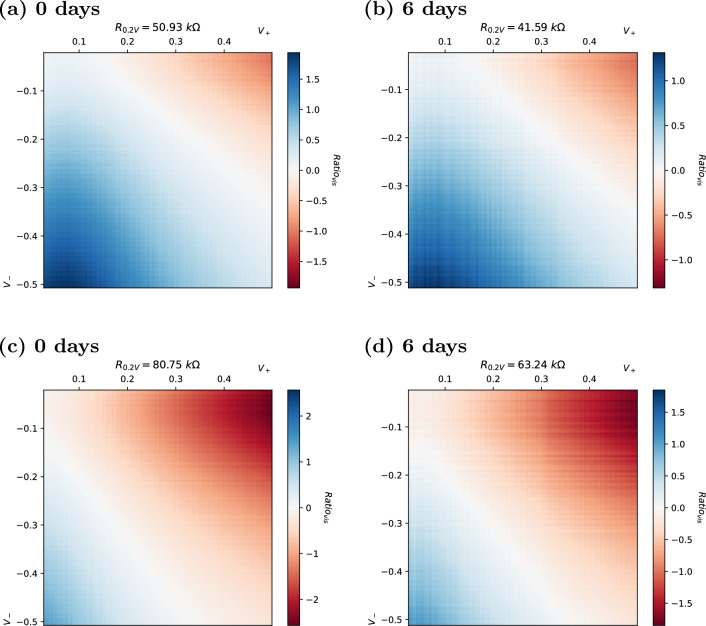
2D heat map of $Ratio_{vis}$ [Eq. (1)] for selected stable cells at 0 days (**a**, **c**) and 6 days (**b**, **d**). Note that for each cell, [20, 20] (**a**, **b**) and [17, 16] (**c**, **d**), there is a small variation in resistance at $+0.2 \, V$ (shown above each plot) after 6 days of ageing, but the $Ratio_{vis}$ response remains distinct.

These responses are also visually stable over longer time periods, as shown in Fig. 5. The figure shows that, whilst there is some shift in the absolute resistance (at 0.2 V, shown above plots), the overall visual similarity between the plots remains strong. This is also fundamental to fingerprinting, as the signature must remain uniquely identifiable to generate a reproducible response.

Initial analysis showed that not all the cells proved to be reliable over the longer-term. The analysis revealed that, on the die characterised, 21 out of 32 cells had an inter-ED less than the minimum inter-ED for every 30-min period in an initial 18-h experiment. This demonstrates that, even after being left to settle and accounting for measurement noise, these devices maintain their characteristic responses and can be identified. Further, these cells remained uniquely identifiable to their original reference response and their response matrix did not become so close to another cell’s such as to be confused for one another.

Further analysis was performed to identify the cells given a settling time of six days and again at thirty days. The sample of I–V characteristics at day 0 was considered the “reference” data, and data sampled at future dates were compared to this. The reliable cells found at six days were assumed as stable and further compared at thirty days. This selection method is similar to that employed in PUFs to identify reliable bits^30^. The Euclidean distance [Eq. (2)] was employed by calculating the distance for each cell in the new data to every cell in the reference dataset. The reference cell with the minimum Euclidean distance was chosen as the best matching cell.

After a six day settling period, 18 out of the 32 cells could be successfully matched to the correct cell in the reference sample (a 56% success rate). This compares to a an expected success rate of $\frac{1}{32}$ (3.1%) for random chance. Further, after a thirty day settling period 21 out of the 32 cells could be successfully matched to the correct cell in the reference sample. Of the cells matched successfully in the six day sample, 16 continued to be correctly matched at thirty days, an 89% success rate. By excluding cells from the six day sample which exhibit excessive linearity (likely to be only acting as a resistor), 11 were successfully matched. Of these 11, 10 were successfully matched at thirty days. The only failed match was clearly identifiable as open circuit behaviour, suggesting its pads may not have made proper contact with the probes. After excluding cells with identifiable error states—high linearity in early testing and open circuit in identifying—100% of cells could be identified correctly after a thirty day period of ageing. This suggests that cells that appear reliably identifiable after a shorter initial observation period will remain identifiable after a longer period.

By increasing the size of the sampled array, or sampling further arrays on a chip, more credibility can be lent to the fingerprinted die being the same as the reference die.

## Comparison with alternative technologies

A comparison of this work with alternative technologies is shown in Table 1.

**Table 1:** Comparison with alternative technologies.

	Serial numbers	Watermark^2^	SRAM PUF^7^	Existing memristor PUF^20^	Memristor fingerprinting
Clone detection	Y	N	Y	Y	Y
Overproduction protection	N	N	Y	Y	Y
Clone resistance	Write-once memory (poor protection)	Comparing watermark—detects only functional clones	SRAM physically clonable, PUF keys may be copied	No known attacks	No known attacks
Key generation	Weak	N	Y	Y	Y
Unique ID	Y	N	Y	Y	Y
Auditable off-chip	N	Y	Y	Y	Y
Simple power characteristic	N	N	N	N	Y
On-chip resources	Read-only memory	Alterations to design	On-chip SRAM	Crossbar RRAM	Standalone RRAM

Because the proposed methodology only depends on a single memristor being addressed at a time on a chip, and only requires the two terminals of the memristor to be powered, hardware attacks on the chip attempting to simulate the responses my be identified. By not powering the chip, except for the memristor, the only power to the chip will be the memristor itself. Any disruption in power by an attack circuit will be detectable in the I–V characteristic and will therefore be identifiable.

In an SRAM PUF a key is generated based on the uncertain initial start-up value of an SRAM memory. The value of an SRAM when power is applied is undefined and can vary between memories due to manufacturing variation. This effect has been previously exploited as a PUF but may be clonable. For example, an SRAM could be intentionally biased at manufacture to identically clone the response of a legitimate chip. Such attacks are an intrinsic issue in digital weak PUFs that rely only on a digital string being reproduced.

In comparison, the memristor-based fingerprinting technique relies not only on the state of the cell (which is analogue as opposed to a single digital value) but also on the varying analogue behaviour over a range of voltages. Taken in total, fingerprinting based on memristors’ analogue responses offers a great improvement in cloning resistance from an attacker with physical access by increasing the barriers to producing a successful clone. As mentioned previously, an attempt to insert an attack to simulate a correct fingerprint response will be identifiable in the response. Because producing a perfect clone of a memristor is difficult there is an inherent increase in difficulty for an attacker as compared to an SRAM PUF.

Most existing memristor-based PUFs depend on a large crossbar memory, or integration with CMOS components in the loop^19–21^. This leads to a higher complexity required to identify devices and therefore leaves more options for attack, as well as making identifying attacks more difficult. In comparison, this work in its simplest form requires only standalone memristors to be integrated on the chip, rather than a crossbar array. It also exploits a different source of variation in memristors by primarily relying on the I–V curve variation, rather than absolute resistances. Because of this reliance, some memristive technologies may need an adaptation of methodology or not be applicable to this type of fingerprint. The $\text {Ta}/{\text {HfO}_{2}}$ memristor used by^22^, for example, exhibits a linear I–V response in the LRS.

When compared to^20^, a memristive crossbar-based PUF which partly exploits voltage dependent resistance behaviour, this work offers improved auditability and general applicability to any given reasonably-reliable memristive technology.

## Conclusions

This paper has outlined a methodology for fingerprinting arrays of standalone memristors, and identifying the fingerprint at later date. This research has applicability to high security applications where a very low area, low cost fingerprint is desired. These fingerprints are based on not only the resistive state of the cells, but also their analogue I–V characteristics. Because the I–V characteristics are more difficult to clone than the resistance of a memristor at a single voltage, a much greater level of protection against cloning is afforded than a static identifier simply programmed into memory. Because of this additional level of uncertainty, a much greater level of security is offered than that against other approaches, such as SRAM PUFs.

By using this methodology an array of 10 reliable memristor cells could be identified correctly as the same chip after an ageing period of 30 days under typical environmental conditions.

Further research may be conducted using this methodology to determine the resistance of the fingerprint to environmental effects (temperature variation) as well as additional ageing. Larger arrays could offer greater variation which may be used to improve the certainty of correct identification. To exploit large arrays it may be advantageous to investigate options for integrating selection logic on-chip to enable these arrays to be characterised off-chip without consuming a large number of pins or requiring microprobing equipment. In doing so, care must be taken to avoid compromising the auditability and simplicity of the approach. Additionally, since this work is based on only testing a $\text {TiO}_x$-based memristor technology, other memristive technologies should also be assessed for potential for I–V based fingerprinting. This could include whether variability remains unique for these technologies, or whether it may be strongly dependent on resistive state.

## Supplementary Information

Supplementary Figures.

## Data Availability

The datasets used and/or analysed during the current study available from the corresponding author on reasonable request.
