# Supplementary material for: A memristor fingerprinting and characterisation methodology for hardware security
Source: Sci Rep. 2023 Jun 9;13:9392. doi: 10.1038/s41598-023-33051-z (PMC10256690; doi:10.1038/s41598-023-33051-z)
Supplement: Supplementary file 1 — Supplementary Figures. [file 41598_2023_33051_MOESM1_ESM.pdf]

A Memristor Fingerprinting and Characterisation Methodology for Hardware Security - Supplementary Information

1 Supplementary Figures

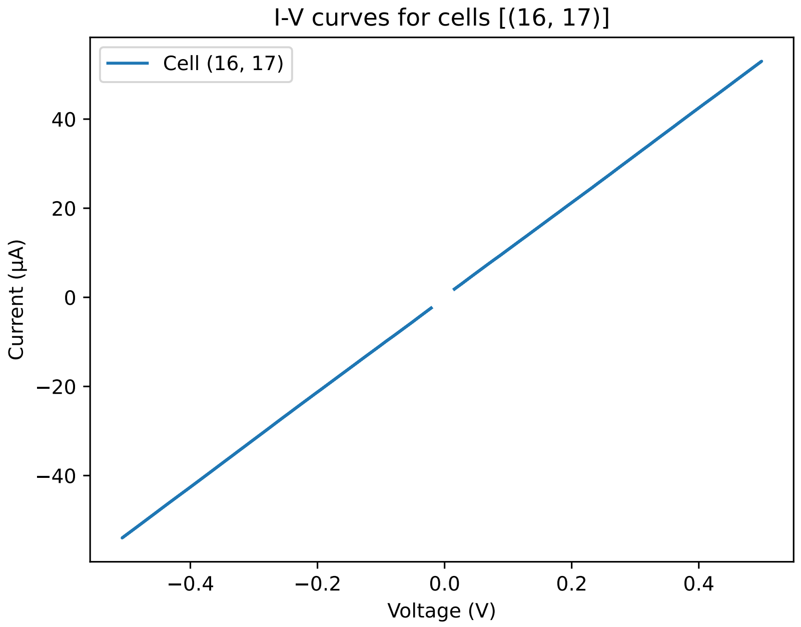

Figure 1: I-V plot for non-functional memristor cell

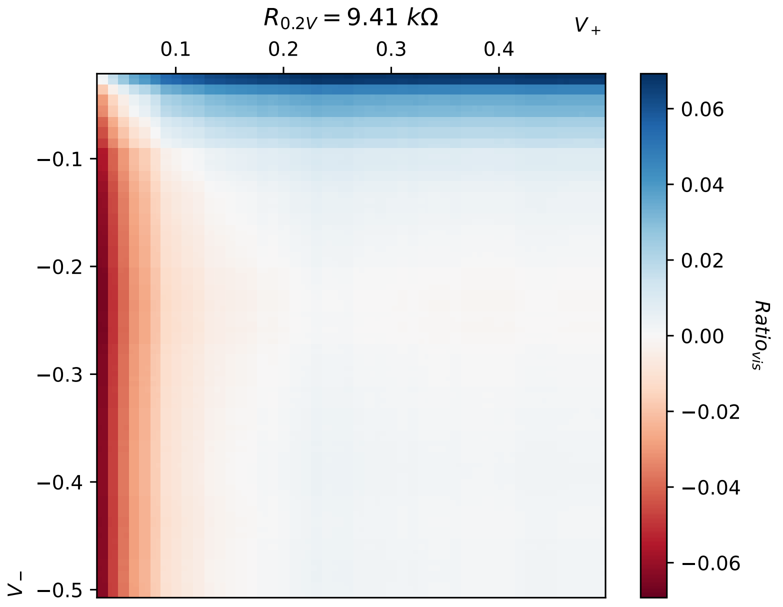

Figure 2: 2D plot of $Ratio_{vis}$ for cell in figure 1

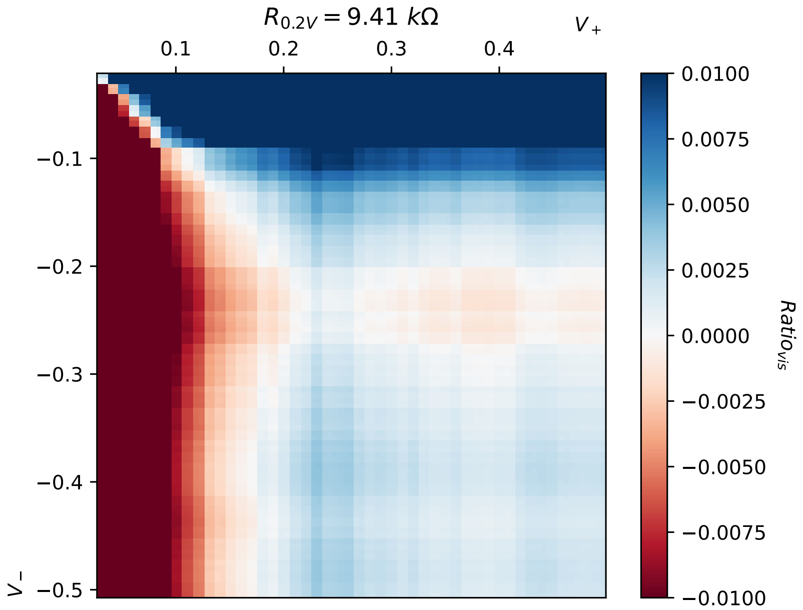

Figure 3: 2D plot of $Ratio_{vis}$ for cell in figure 1 with a 0.01 scale limit
